# What are the inpatient and day case costs following primary total hip replacement of patients treated for prosthetic joint infection: a matched cohort study using linked data from the National Joint Registry and Hospital Episode Statistics

**DOI:** 10.1186/s12916-020-01803-7

**Published:** 2020-11-18

**Authors:** Kirsty Garfield, Sian Noble, Erik Lenguerrand, Michael R. Whitehouse, Adrian Sayers, Mike R. Reed, Ashley W. Blom

**Affiliations:** 1 Health Economics at Bristol, Population Health Sciences, Bristol Medical School, University of Bristol, Bristol, UK; 2 Bristol Trials Centre, Bristol Medical School, University of Bristol, Bristol, UK; 3 Musculoskeletal Research Unit, Translational Health Sciences, Bristol Medical School, University of Bristol, Bristol, UK; 4 National Institute for Health Research Bristol Biomedical Research Centre, University Hospitals Bristol NHS Foundation Trust and University of Bristol, Bristol, UK; 5 Department of Trauma and Orthopaedics, Wansbeck General Hospital, Northumbria Healthcare NHS Foundation Trust, Ashington, UK

**Keywords:** Prosthetic joint infection, Surgical site infection, Hip replacement, Orthopaedics, Costs, Cohort study, Hospital Episode Statistics, National Joint Registry

## Abstract

**Background:**

Prosthetic joint infection (PJI) following total hip replacement (THR) surgery is a serious complication that negatively impacts patients’ lives and is financially burdensome for healthcare providers. As the number of THRs increases, so does this financial burden. This research estimates the economic burden with respect to inpatient and day case hospital admissions for patients receiving revision surgery for PJI following primary THR.

**Methods:**

In this matched cohort study, the National Joint Registry for England, Wales, Northern Ireland and the Isle of Man (NJR) was used to identify patients. Patients revised for PJI with a one- or two-stage revision following THR and patients not revised for PJI were matched on several characteristics using exact and radius matching. Hospital inpatient and day case healthcare records from the English Hospital Episode Statistics database were obtained for 5 years following the identified patient’s primary THR. UK national unit costs were applied to hospital admissions and the 5-year total cost was estimated. A two-part model (Probit and generalised linear model) was employed to estimate the incremental difference in costs between those revised and not revised for PJI.

**Results:**

Between 2006 and 2009, 1914 revisions for PJI were identified in the NJR. The matching resulted in 422 patients revised for PJI and 1923 matches not revised for PJI who were included in the analysis. The average cost of inpatient and day case admissions in the 5 years following primary THR was approximately £42,000 for patients revised for PJI and £8000 for patients not revised for PJI. The difference in costs over the 5 years was £33,452 (95% CI £30,828 to £36,077; *p* < 0.00).

**Conclusions:**

In the 5 years following primary THR, patients who develop PJI and have revision surgery cost approximately £33,000 (over 5-fold) more than patients not revised for PJI based on their hospital inpatient and day case admissions alone. The total burden of PJI is likely to be much higher when also considering outpatient, primary and community care costs. This highlights the need to find both ways to reduce the incidence of PJI following THR and cost-effective treatment strategies if PJI occurs.

## Background

Total hip replacement (THR) is a cost-effective treatment that has been shown to relieve pain, restore function and enhance quality of life [1, 2]. Approximately 58% of THRs will last for 25 years or more [3]; however, a small percentage of patients will develop a periprosthetic joint infection (PJI), as a complication of their THR [4]. The risk of revision for PJI is estimated at 0.4% following primary THR and 1.6% following aseptic revision THR [4]. PJI is a serious and debilitating complication that is likely to negatively impact morbidity and quality of life and increase the risk of mortality [5–9].

Treatment options for PJI of the hip include surgical debridement and implant retention with or without a modular exchange, one- or two-stage revision arthroplasty, excision or amputation. When compared to primary THR and aseptic revision, revision procedures for PJI are associated with longer operating times, increased blood loss and more complications [10]. The burden associated with PJI is also exacerbated by high readmission rates, costly repeat procedures, extended hospital stays, increased use of hospital outpatient services and prolonged use of intravenous and oral antibiotics [10–15].

The burden of primary and revision THR is increasing worldwide. Between 2003 and 2013, statistically significant increases in the life-time risk of THR were estimated in Australia, Denmark, Finland, Norway and Sweden [16]. In the USA, between 2005 and 2030, demand for primary and revision THR was projected to rise by 174% and 137%, respectively [17]. While in England and Wales, between 2012 and 2030, demand for primary and revision THR was projected to rise by 134% and 31%, respectively [18]. In England and Wales, the number of hip revisions performed due to PJI is rising which is increasing the economic burden of managing this complication [4]. Several studies have estimated the costs associated with treatment for hip PJI [10, 19, 20]. In the USA, for a single episode of care, the direct cost of treating PJI has been estimated as approximately US$100,000 [10, 19], with the overall lifetime treatment cost for a 65-year-old estimated at US$390,806 [20]. In the UK, the mean total cost for revision for PJI surgery in 2007/2008 was estimated at £21,937 [21]. At the hospital level, research has found that reimbursement for revision arthroplasty for PJI does not meet the cost [13, 21], suggesting an increased financial burden on treating hospitals.

The objective of this study is to estimate the cost to the English National Health Service (NHS) of inpatient and day case admissions, in the 5 years following primary THR, of patients who develop PJI of the hip and undergo a one- or two-stage revision compared to those who do not, using linked National Joint Registry (NJR) and Hospital Episode Statistics (HES) data.

## Methods

### Study design and setting

This matched cohort study utilised data from the National Joint Registry for England, Wales, Northern Ireland and the Isle of Man (NJR), which was linked to inpatient and day case admission data from the Hospital Episode Statistics (HES) between April 1, 2003, and December 1, 2014. HES data includes data on inpatient and day case admissions in England funded by the English NHS, as such, the analysis was limited to patients receiving NHS-funded treatment in England.

### Study population

In this study, we aimed to compare the inpatient and day case costs of patients who underwent one- or two-stage revision THR for PJI following their primary THR (revised PJI patients hereinafter) compared to matched patients whose THR was either not revised or revised for reasons not related to PJI (comparator patients hereinafter).

Patients were eligible for inclusion in the revised PJI group if one of the indications for revision was recorded as infection by clinicians in the NJR at the time of revision (more than one indication can be selected); they received a one-stage revision or at least part one of a two-stage revision for PJI between 2006 and 2009; the surgery was the first revision for PJI on the index side (the index side refers to the hip side that is included in the analysis, for revised PJI patients it is the side with the hip that was treated for infection); their primary THR could be identified in the NJR; they did not have revision surgery for PJI on the non-index side during the 5 years following THR surgery for their index side; the revision surgery for PJI was within 5 years of their primary; they had complete matching variables and their NJR records could be linked to HES.

Patients were eligible for inclusion in the comparator group if they had a primary THR between the dates of the primary THRs of revised PJI patients; they did not have revision for PJI on their index side (for comparator patients, the index side is the side with the hip that had a primary THR during the period of revised PJI patients THRs) reported in the NJR data (available until 2009); they did not have revision surgery for PJI on the non-index side during the 5 years following THR surgery for their index side; they had complete matching variables and their NJR records could be linked to HES. Comparator patients could have had revision surgery for indications other than PJI. Comparator patients may also have developed a PJI and received alternative treatments such as antibiotic suppression.

Once eligible patients were identified they were matched using a combination of exact and radius matching with a matching ratio of 1 revised PJI patient to 5 comparator patients, without replacement. To maximise the sample size, where less than 5 comparator patients were identified, the revised PJI patient and matching comparator patient(s) were still included. Matching variables were selected from patient characteristics and primary THR surgery factors that previous research suggests potentially impact the likelihood of PJI following THR [22, 23]. The matching process incorporated exact matching for some variables (sex, ASA grade, type of hip replacement (total or resurfacing), hospital) and radius (close) matching for variables where we were unlikely to find exact matches (date of primary THR, age). We allowed a radius of plus or minus 1 year for the date of primary THR and plus or minus 10 years for age.

### Identification of resource-use and estimation of cost

For all patients, inpatient and day case admissions (not limited to orthopaedics admissions) reported in HES for the 5 years following their primary THR was cleaned and processed through the HRG4+ 2014/2015 Reference Costs Grouper [24] to obtain Healthcare Resource Groups (HRG’s). An HRG is a group of clinically alike treatments that use similar levels of healthcare resource. Cleaning included identifying and removing duplicate records and reformatting HES records to enable them to be processed by the Grouper. NHS reference costs were used to estimate costs; reference costs are based on the average unit costs of NHS providers [25]. HRGs are costed by applying reference costs to the core HRG, unbundled HRGs and excess bed days. Spell-level reference costs, where spell refers to a single hospital stay from admission to discharge, were applied to each spell HRG using NHS reference costs [25]. Where the Grouper did not provide an HRG, weighted average costs of adult HRGs by admission type (elective, non-elective short/long stay, regular day/night, day case) were applied. Costs of each HRG spell were then summed to estimate 5-year costs following primary THR.

### Analysis

All statistical analyses were performed in Stata 15.1 (StataCorp LLC, College Station, TX [26]). Multiple model specifications were explored and compared using, but not limited to, histograms, quantile-quantile and percentile plots of deviance residuals and Akaike’s information criterion. A two-part model [27, 28], which accounted for clustering of revised PJI and comparator patients within their matching group, was employed using the twopm Stata command [29] to estimate the difference in number of stays and costs. To account for excess zeros, in the first part of the model, a Probit model was used to estimate the probability of total costs equalling zero. In the second part, a generalised linear model was used to assess the distribution of costs in revised PJI and comparator patients who had at least one inpatient or day case admission, with an identity link function and gamma distribution to account for the positively skewed distribution of costs. Age, sex, ASA grade, diagnosis of osteoarthritis, operation date, Charlson Comorbidity Index, bearing surface (metal-on-polyethylene, metal-on-metal, ceramic-on-polyethylene, ceramic-on-ceramic, metal-on-ceramic/ceramic-on-metal) and procedure (cemented, uncemented, hybrid, reverse-hybrid, resurfacing) were controlled for within the model. To account for the intragroup correlation of patients within matched groups, a variable indicating the matched group was included as a variance estimator cluster option in the model. Further information on the model specification is provided in Additional File 1.

## Results

**Fig. 1:**
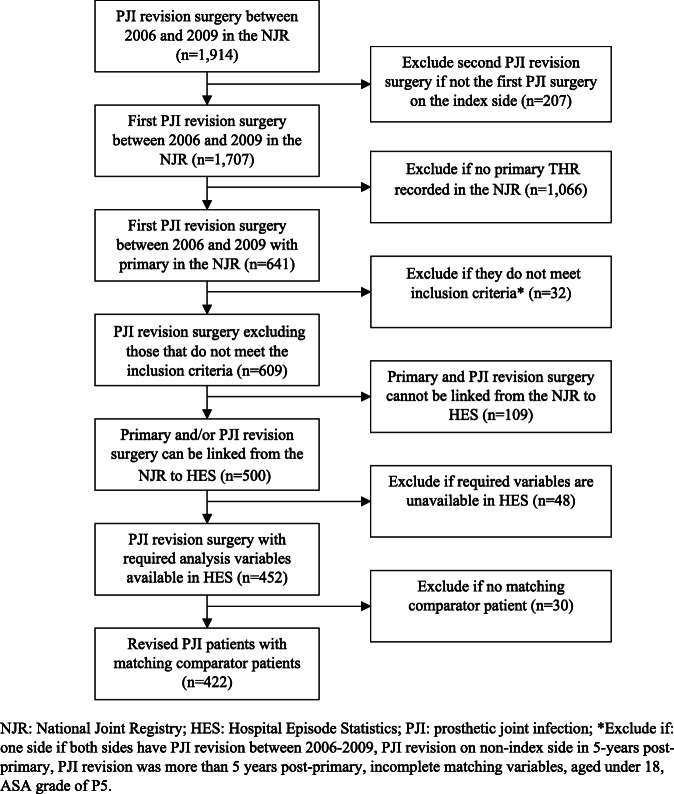
Identification of patients revised for PJI

**Fig. 2:**
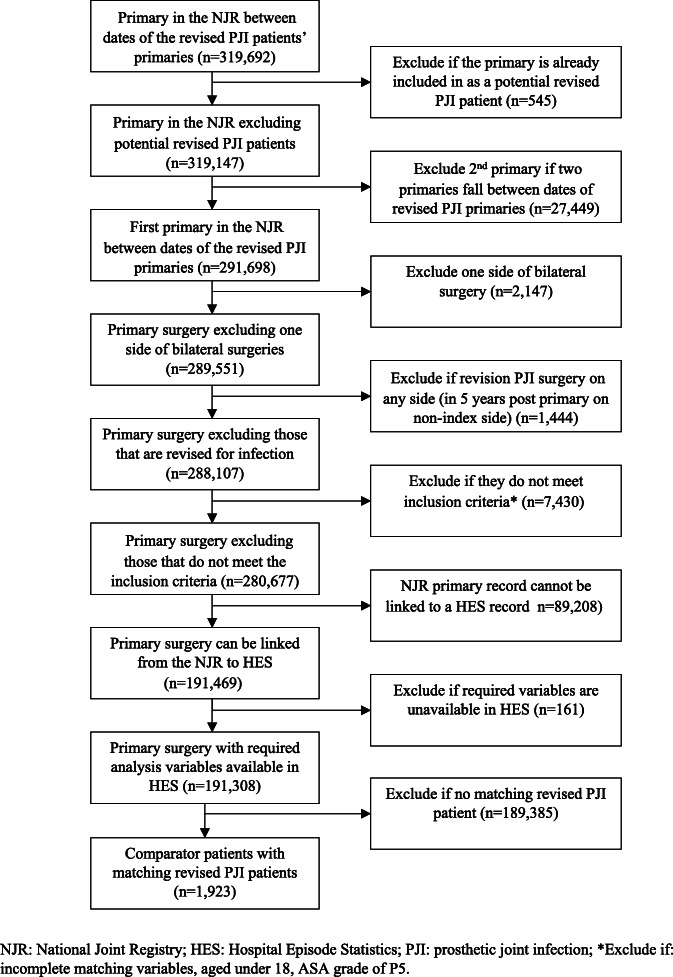
Identification of comparator patients

The identification of revised PJI and comparator patients is presented in Figs. 1 and 2, respectively. Between 2006 and 2009, 1914 revisions for prosthetic joint infection were reported in the NJR. For 1707 (89%), the surgery was the patient’s first one- or two-stage revision THR for PJI on the index side. Of these patients, 609 met the pre-defined inclusion criteria for the revised group, and for 500, we could link the primary and/or revision surgery in the NJR to HES. Patients not revised for PJI were identified by locating any primary THR, in the NJR, that occurred between the dates of primary THRs for revised PJI patients. Of 319,692 THRs identified in the NJR, 191,469 met the comparator patient inclusion criteria and could be linked to HES.

Exact and radius matching was performed on 452 patients revised for PJI and 191,308 patients not revised for PJI. Of these, 422 revised PJI and 1923 comparator patients were matched and included in the analysis. Five matching comparator patients were found for 85% of revised PJI patients. Of the remaining 15%, four matching comparator patients were found for 3%, three matching comparator patients were found for 2%; two matching comparator patients were found for 3% and one matching comparator patient was found for 3%. As expected, there was balance between revised and comparator patients for variables that were matched on. Other variables were moderately balanced between the two groups and were subsequently adjusted for in the analysis model.

Patient characteristics at primary THR are presented in Table 1. Balance between revised PJI and comparator groups was achieved through exact and radius matching. The mean age was 66 (range 21 to 95) in the revised PJI group and 67 (range 23 to 92) in the comparator group. Forty-five per cent and 46% of patients were female in the revised PJI and comparator groups, respectively. Most patients had an ASA grade of P2 (revised PJI = 71%; comparator = 73%). Moderate balance was observed between groups for the Charlson Comorbidity Index, which was not included in the matching but was controlled for in the model. In both groups, most patients had a Charlson score of zero (revised PJI = 65%; comparator = 74%). Ninety-four per cent of revised PJI and 97% of comparator patients had an osteoarthritis diagnosis at primary THR. Most patients received either a cemented (revised PJI = 39%; comparator = 41%) or uncemented (revised PJI = 37%; comparator = 35%) primary THR, with a metal-on-plastic bearing type (revised PJI = 60%; comparator = 61%).

**Table 1:** Characteristics of matched patients revised and not revised for PJI following primary THR

	Revised PJI group (***n*** = 422)	Comparator group (n = 1923)	
	Number (%)	Number (%)	
Date of primary—range	16/05/03–02/12/09	28/04/03–01/12/09	
Age—mean (range)	66 (21–95)	67 (23–92)	
Female	191 (45)	891 (46)	
Osteoarthritis diagnosis	398 (94)	1862 (97)	
ASA grade			
P1	69 (16)	302 (16)	
P2	298 (71)	1399 (73)	
P3	55 (13)	222 (12)	
Charlson			
0	275 (65)	1415 (74)	
1	97 (23)	333 (17)	
2	31 (7)	104 (5)	
3 or above	19 (5)	71 (4)	
Procedure			
Cemented	164 (39)	782 (41)	
Uncemented	158 (37)	668 (35)	
Hybrid/reverse hybrid	64 (15)	324 (17)	
Resurfacing	36 (9)	149 (8)	
Bearing type			
Metal-on-plastic	254 (60)	1179 (61)	
Metal-on-metal	99 (23)	354 (18)	
Ceramic-on-ceramic	41 (10)	199 (10)	
Ceramic-on-plasticmetal-on-ceramic/ceramic-on-metal	28 (7)	191 (10)	
Matches per revised PJI patient			
5 matching comparator patients	358 (85)		
4 matching comparator patients	13 (3)		
3 matching comparator patients	9 (2)		
2 matching comparator patients	12 (3)		
1 matching comparator patients	30 (7)		

Assessment of model specification suggested the model fitted well (see Additional File 1). On average, patients revised for PJI with a one- or two-stage THR had eight admissions during the 5 years post-primary. Comparator patients had three admissions on average, including a large proportion (24%) who did not have any inpatient or day case admissions in the 5 years following THR. The Grouper provided HRGs for more than 98% of spells. Using spell-level costing, we found that the average cost of inpatient and day case admissions in the 5 years following primary THR was £41,633 (95% CI £39,079 to £44,187) for patients revised for PJI and £8181 (95% CI £7614 to £8748) for patients not revised for PJI, a difference in costs of £33,452 (95% CI £30,828 to 36,077; *p* < 0.00) (Table 2). Annual costs (Table 2) show the adjusted difference in costs diminished over the 5 years following primary THR.

**Table 2:** Average total and annual inpatient and day case hospital admission costs over the 5 years following THR, by revised PJI and comparator patients

	Revised PJI group (***n*** = 422)	Comparator group (***n*** = 1923)	Adjusted difference in costs (£) (95% confidence interval)
	Adjusted cost (£)	Adjusted cost (£)	
	Mean (SE)	Mean (SE)	
1st year post-primary	14,686 (816)	1959 (111)	12,727 (11,094 to 14,360)
2nd year post-primary	10,575 (682)	1503 (91)	9071 (7719 to 10,424)
3rd year post-primary	6974 (580)	1512 (97)	5462 (4306 to 6618)
4th year post-primary	5168 (501)	1584 (131)	3584 (2611 to 4557)
5th year post-primary	4427 (431)	1568 (101)	2859 (1999 to 3720)
**Total over 5 years**	**41,633 (1303)**	**8181 (289)**	**33,452 (30,828 to 36,077)**

Marginal means after adjusting for excess zero; adjusted for age, sex, ASA grade, diagnosis of osteoarthritis, operation date, Charlson Comorbidity Index, bearing surface and procedure

## Discussion

### Main findings

This study has shown that in the 5 years following their primary THR, patients who have one- or two-stage revision THR for PJI have more hospital admissions than patients who do not receive revision THR for PJI, at an estimated additional cost of £33,452. The findings from this study support the hypothesis that patients who are revised for PJI following THR cost significantly more than patients who are not revised for PJI.

### Comparison to relevant literature

Several studies have attempted to estimate the financial burden of PJI following primary THR. The cost of initial treatment for PJI with a 2-stage revision was estimated at 21,937 GBP in the UK in 2007/2008 [21] and 60,394 euros (71,953 GBP (inflated [30] and converted using a 2016 purchasing power parity [31])) in Italy using PJI surgeries identified between 2001 and 2006 [32]. This study supports the findings from previous matched studies where patients who are treated for PJI following THR have increased healthcare costs when compared to patients not treated for PJI [14, 33]. In 2016, Kapadia and colleagues undertook a matching study at a single centre, to estimate healthcare costs, length of hospital stay and number of readmissions for patients who develop deep PJIs, compared to patients who did not develop a PJI within 1 year following primary THR [14]. Based on 16 infected and 32 uninfected patients, they found that over the first year following primary THR, infected patients cost 62,964 USD (48,568 GBP (inflated [30] and converted using a 2016 purchasing power parity [31])) more than uninfected patients [14]. This represents a larger difference than estimated in our analysis; however, the results are not directly comparable as this study incorporated a wider range of costs, such as outpatient care. Using data from a single hospital, between 2005 and 2011, González-Vélez and colleagues also performed a matching study comparing 81 infected cases and 81 uninfected controls identified at a public hospital in Spain [33]. Over 1 year following hip replacement, they found that direct hospital costs were on average 134% higher and length of admissions 176% longer for patients who developed a surgical site infection [33]. In comparison, we found that during the first year following THR, costs for revised PJI patients were over 13 times higher than those in the comparator arm. As above, the results are not directly comparable as González-Vélez and colleagues only incorporated re-admissions due to infections whereas out analysis incorporates inpatient and day case stays for any cause [33].

### Strengths and weaknesses

We explored using different time frames to identify patients treated for infection with a one- or two-stage THR. The decision to only include patients in this study who had revision for PJI between 2006 and 2009, which meant that not only were we able to have a long follow-up period of years following primary THR but it increased the likelihood that the primary THRs were recorded in the NJR which began collecting data in 2003. This method resulted in a large sample size when compared to other studies exploring the costs of PJI treatment.

Patients were included in the revised PJI group if they had a one- or at least part one of a two-stage revision for PJI recorded in the NJR, and comparator patients were identified as those not receiving a one- or two-stage revision for PJI. A minority of comparator patients may have developed a PJI and have had alternative treatments. As a result, our conclusions on the cost burden does not compare infected with uninfected patients but compares those revised for PJI with a one- or two-stage revision compared to those not revised for PJI. The indication for revision in the studied dataset is defined at the time of revision. The incidence of revision for PJI may therefore be an underestimate as microbiology from samples taken intraoperatively and other intraoperative test results that may influence the opinion of the treating surgeon would not always be available at the time the indication was selected. Equally, the indication for surgery is not therefore influenced by potential contaminants on microbiology samples taken intraoperatively which may increase the number of revisions attributed to PJI when this was not the case. There could also be differences observed between different types of THR with misdiagnosis of PJI being a recognised phenomenon in adverse reaction to debris in THR [34].

Access to the large number of patients within the NJR meant that 94% of revised PJI patients were matched to comparator patients using exact and radius matching. Other matching methods were considered. Exact and radius matching was chosen over propensity score matching due to the match being performed ex ante, allowing us to estimate the costs of matched patients post matching. Propensity score matching would have required 5-year costs to be estimated prior to matching, which would have required the cleaning and costing of the HES records of over 190,000 patients. To maximise the sample size, revised PJI patients were included as long as at least one matching comparator patient was available. Although this meant that a one revision PJI to five comparator patients ratio was not achieved for all patients, exact matching variables remained well balanced, suggesting matching performed well.

The majority of variables are well-completed in the NJR; therefore, we excluded few patients due to missing matching variables. Body mass index was the exception, due in part to it not being included in earlier data collection forms. Body mass index, a known risk factor for infection [23], was therefore excluded from the regression analysis, which is a limitation of the analysis. The richness of the NJR dataset meant that all other known confounders were matched for or controlled for within the regression.

All inpatient and day case admissions were included in the analysis, not just those related to the hip. We included admissions for all indications as PJI may affect other areas of patients’ lives, leading to admissions for reasons not directly related to the PJI.

In this study, while we have estimated the burden of PJI with respect to inpatient and day case admissions, if the outpatient, primary and community care and prescribed medication costs were also estimated, it is likely that the total cost of healthcare for treating PJI would be much higher. In addition, as HES includes hospital admissions at NHS hospitals in England, the cost of admissions funded by the NHS outside of England or in private facilities was not incorporated.

## Conclusion

In the 5 years following THR, patients who have a revision THR for PJI cost approximately £33,000 (over 5-fold) more than patients not revised for PJI, based on their hospital admissions alone. This research, which to our knowledge has the largest sample size of studies in this area, adds to current evidence that PJI of the hip following THR represents a significant financial burden to healthcare commissioners/payers. This highlights the need to find ways to reduce the incidence of PJI following THR and to establish cost-effective treatment strategies if PJI occurs.

## Supplementary information

**Additional file 1: Summary of model specification**. **Figure S1.** Deviance residuals and percentile plot of the generalised linear model part of the 5-year cost model. **Figure S2.** Proportion of patients with zero costs over the five years and each year post primary THR, by revised PJI and comparator groups.

## Data Availability

Data are accessible via application to the National Joint Registry Research Sub-Committee.

## Abbreviations

HES: Hospital Episode Statistics
HRG: Healthcare Resource Group
NHS: National Health Service
NJR: National Joint Registry for England, Wales, Northern Ireland and the Isle of Man
PJI: Prosthetic joint infection
THR: Total hip replacement
