# Supplementary material for: What are the inpatient and day case costs following primary total hip replacement of patients treated for prosthetic joint infection: a matched cohort study using linked data from the National Joint Registry and Hospital Episode Statistics
Source: BMC Med. 2020 Nov 18;18:335. doi: 10.1186/s12916-020-01803-7 (PMC7672908; doi:10.1186/s12916-020-01803-7)

**Additional File 1**

**Summary of model specification**

Independent analyses were performed for total 5-year cost, annual costs and the number of stays. Due to the large proportion of excess zeros (particularly in the comparator group and for annual costs) two-part models were deemed most appropriate. In the first part of the two-part models, a logit model was specified to predict the probability of any inpatient/day case costs/stays. In the total 5-year cost model this was specified as:

*Prob(5_year_cost_i_>0) = β_0_ + β_1_group_i_ + β_2_age_i_ + β_3_sex_i_ + β_4_ASA_i_ + β_5_charlson_i_ + β_6_osteoarthritis_i_ +* β_7_operation_date_i_ *+ β_8_procedure_i_ + β_9_bearing_i_*

where *5_year_cost* was the total all-cause inpatient and day case costs over the five years following primary THR. Variable *group_i_* represents whether patient i was in the revised PJI or comparator group. All other variables were captured at primary THR. Patient characteristics included age (*age_i_*) and sex (*sex_i_*). Variables reflecting patients’ health status included ASA grade (*ASA_i_*), Charlson Comorbidity Index group (*charlson_i_*) and osteoarthritis diagnosis (*osteoarthritis_i_*). The date of the primary THR was represented by (*operation_date_i_*). Variables *procedure_i_* and bearing_i_ relate to the procedure and bearing type used in patient i’s primary THR.

In the second part of the two-part model, a generalised linear model was implemented with an identity link function and gamma distribution to account for the positively skewed distribution of costs/stays. This model predicts the level of inpatient/day case costs/stays conditional on having inpatient/day case costs/stays. In the total 5-year cost model this was specified as:

*5_year_cost_i_|5_year_cost_i_>0 = β_0_ + β_1_group_i_ + β_2_age_i_ + β_3_sex_i_ + β_4_ASA_i_ + β_5_osteoarthritis_i_ + β_6_operation_date_i_ + β_7_charlson_i_ + β_8_procedure_i_ + β_9_bearing_i_*

The two-part models were implemented in Stata using the *twopm* command (29). The margins command was subsequently used to obtain marginal means after adjusting for excess zeros. The case/control effect was included as a random effect in the model using a clustered sandwich estimator (vce(cluster) option in Stata) to obtain robust variance estimate that adjusts for similarity of patients within clusters (within-cluster correlation induced by matched revision PJI and comparator patients). In Figure S1, the histogram of the deviance residuals, showing the distribution of residuals for all observations and a percentile plot suggest the model fitted well.

Figure S1: Deviance residuals and percentile plot of the generalised linear model part of the 5-year cost model


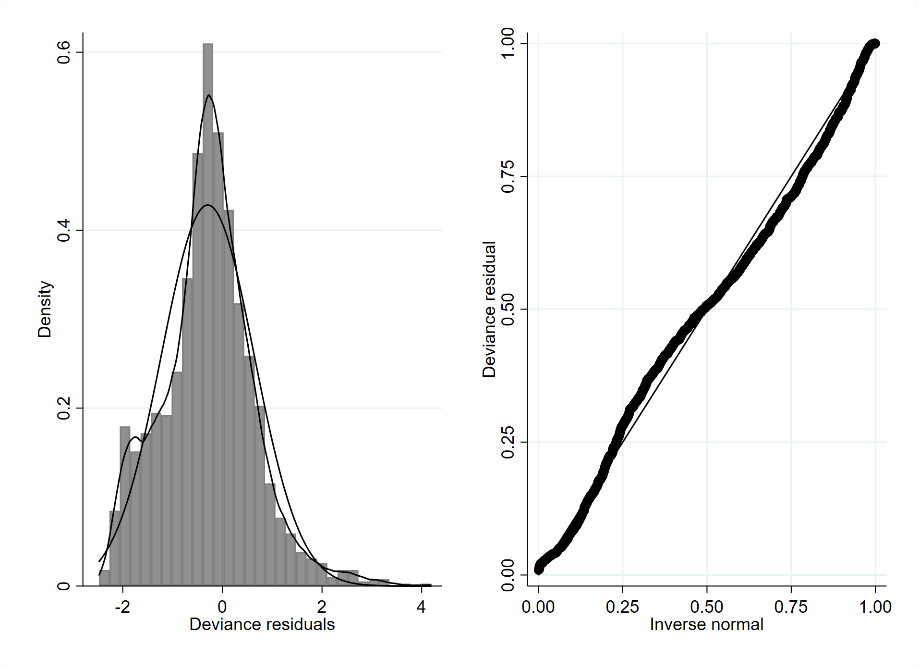


Figure S2: Proportion of patients with zero costs over the five years and each year post primary THR, by revised PJI and comparator groups


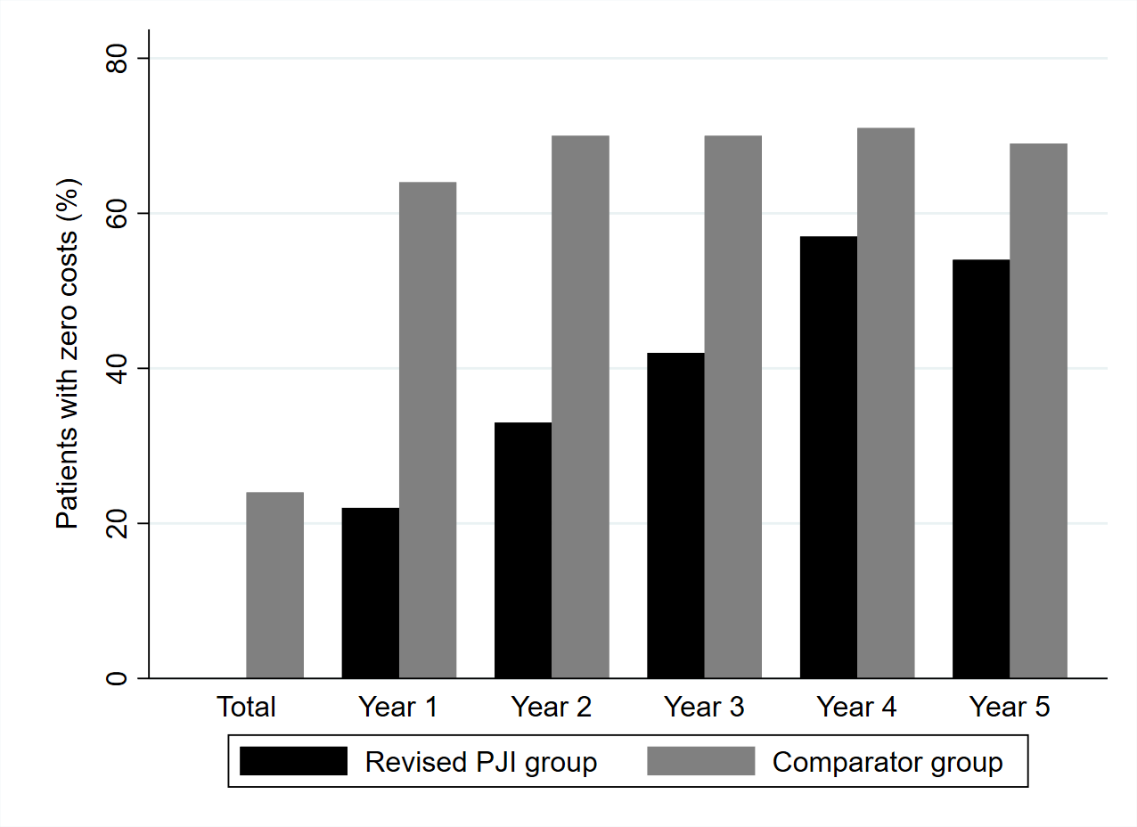

Supplement: Supplementary file 1 — Additional file 1: Summary of model specification. Figure S1. Deviance residuals and percentile plot of the generalised linear model part of the 5-year cost model. Figure S2. Proportion of patients with zero costs over the five years and each year post primary THR, by revised PJI and comparator groups. [file 12916_2020_1803_MOESM1_ESM.docx]
